# Increased Expression of Multiple Co-Inhibitory Molecules on Malaria-Induced CD8^+^ T Cells Are Associated With Increased Function Instead of Exhaustion

**DOI:** 10.3389/fimmu.2022.878320

**Published:** 2022-07-07

**Authors:** Johannes Brandi, Mathias Riehn, Alexandros Hadjilaou, Thomas Jacobs

**Affiliations:** ^1^ Protozoa Immunology, Bernhard Nocht Institute for Tropical Medicine, Hamburg, Germany; ^2^ German Center for Infection Research Deutsches Zentrum für Infektionsforschung (DZIF), Partner Site Hamburg-Lübeck-Borstel-Riems, Hamburg, Germany; ^3^ Klinik und Poliklinik für Neurologie, Universitätsklinikum Hamburg-Eppendorf, Hamburg, Germany; ^4^ Institut für Neuroimmunologie und Multiple Sklerose, Zentrum für Molekulare Neurobiologie Hamburg, Universitätsklinikum Hamburg-Eppendorf, Hamburg, Germany

**Keywords:** malaria, co-inhibitory molecules, CD8, immune regulation, LAG-3

## Abstract

Activated cytotoxic CD8^+^ T cells can selectively kill target cells in an antigen-specific manner. However, their prolonged activation often has detrimental effects on tissue homeostasis and function. Indeed, overwhelming cytotoxic activity of CD8^+^ T cells can drive immunopathology, and therefore, the extent and duration of CD8^+^ T cell effector function needs to be tightly regulated. One way to regulate CD8^+^ T cell function is their suppression through engagement of co-inhibitory molecules to their cognate ligands (e.g., LAG-3, PD-1, TIM-3, TIGIT and CTLA-4). During chronic antigen exposure, the expression of co-inhibitory molecules is associated with a loss of T cell function, termed T cell exhaustion and blockade of co-inhibitory pathways often restores T cell function. We addressed the effect of co-inhibitory molecule expression on CD8^+^ T cell function during acute antigen exposure using experimental malaria. To this end, we infected OT-I mice with a transgenic *P. berghei* ANKA strain that expresses ovalbumin (PbTG), which enables the characterization of antigen-specific CD8^+^ T cell responses. We then compared antigen-specific CD8^+^ T cell populations expressing different levels of the co-inhibitory molecules. High expression of LAG-3 correlated with high expression of PD-1, TIGIT, TIM-3 and CTLA-4. Contrary to what has been described during chronic antigen exposure, antigen-specific CD8^+^ T cells with the highest expression of LAG-3 appeared to be fully functional during acute malaria. We evaluated this by measuring IFN-γ, Granzyme B and Perforin production and confirmed the results by employing a newly developed T cell cytotoxicity assay. We found that LAG-3^high^ CD8^+^ T cells are more cytotoxic than LAG-3^low^ or activated but LAG-3^neg^ CD8^+^ T cells. In conclusion, our data imply that expression of co-inhibitory molecules in acute malaria is not necessarily associated with functional exhaustion but may be associated with an overwhelming T cell activation. Taken together, our findings shed new light on the induction of co-inhibitory molecules during acute T cell activation with ramifications for immunomodulatory therapies targeting these molecules in acute infectious diseases.

## Introduction

In malaria, following a bite from a mosquito infected with the *Plasmodium* parasite, the sporozoite travels *via* the blood to the liver. After replication in the liver, referred to as liver stage, the infection ensues into the blood stage with intraerythrocytic parasite replication. During the short liver stage, CD8^+^ T cells recognize and kill infected hepatocytes in an antigen-specific manner through the engagement on MHC class I molecules presenting parasite antigens (1–3). The function of CD8^+^ T cells during the blood stage is, however, less clear and limited due to the absence of MHC class I molecules on erythrocytes. However, recently it was shown that CD8^+^ T cells can directly lyse infected MHC class I expressing reticulocytes in *P. vivax* malaria (4, 5). The blood stage is characterized by strong activation of CD4^+^ and CD8^+^ T cells, which produce pro-inflammatory cytokines (6, 7) that activate phagocytic cells (8), resulting in clearance of infected erythrocytes (9). Dysregulated production of cytokines and effector molecules, however, can contribute to severe malaria (10–13).

Interestingly, in experimental (14, 15) and human malaria (16, 17), multiple co-inhibitory molecules are upregulated on T cells. Subsets of malaria-specific CD8^+^ T cells co-express LAG-3 and PD-1 as well as other molecules associated with T cell exhaustion, for instance, T-cell immunoglobulin and mucin-domain containing-3 (TIM-3) (18), T cell immunoreceptor with Ig and ITIM domains (TIGIT) (19), cytotoxic T-lymphocyte-associated protein 4 (CTLA-4) (20) and CD39 (21, 22). These molecules were shown to be expressed on exhausted T cells in chronic viral infections (23) and in the tumor environment (24–26). Prolonged antigen exposure drives T cell exhaustion (27), which protects the organ’s function and integrity from immunopathology, but can favor viral persistence or immune evasion of malignant cells. T cell exhaustion is a functional adjustment mandatory for long-term viral persistence, leading to a T cell activation level that preserves tissue homeostasis but ensures pathogen control (28, 29).

In chronic viral infection and cancer, blockade of co-inhibitory molecules revokes T cells exhaustion and restores functionality, which is already used clinically in cancer immunotherapy (30). In contrast to oncology or chronic infections, in acute infectious diseases, the function of co-inhibitory molecules is less well defined.

Therefore, we addressed whether the expression of co-inhibitory molecules on malaria-specific CD8^+^ T cell is associated with a decreased function and if the expression level and/or the co-expression of co-inhibitory molecules is associated with a gradual decline in T cell function. We tested this hypothesis using *P. berghei* ANKA (PbA) infection to determine the phenotype and function of activated antigen-specific CD8^+^CD44^+^ T cells not expressing LAG-3 (LAG-3^neg^), expressing low levels of LAG-3 (LAG-3^low^) or high levels of LAG-3 (LAG-3^high^). In conclusion, we have shown that in acute malaria CD8^+^ T cells expressing high levels of multiple co-inhibitory molecules are highly activated, produce more cytokines and are more cytotoxic compared to their counterparts with less or no co-inhibitory molecules.

## Materials and Methods

### Animal Experiments and Infection

C57BL/6J mice and OT-I mice were bred at the animal facility of the BNITM in Hamburg, Germany. All animal experiments were performed according to German law and registered with the “Behörde für Justiz und Verbraucherschutz” under the license N048/2020. Mice were infected with *P. berghei* ANKA. To obtain consistent infections, pre-experimental mice were first injected i.p. with stabilate solution (blood from previously infected mice with 15% glycerol, 2.1% sorbitol in PBS for long-term liquid nitrogen storage). After 5-7 days, blood was taken from the pre-experimental mice to infect mice for the experiment with 1x10^5^ infected red blood cells (iRBC). *Plasmodium berghei* ANKA expressing OVA (PbTG), which was used as indicated was generated by Lundie et al. (31).

### Cell Isolation

Spleens were sieved through a 100 µm cell strainer. Strainers were washed with wash buffer (PBS containing 2% FCS and 1mM EDTA). The cell suspension was centrifuged at 400g for 5 minutes (min) at 4°C. The supernatant was discarded, 20 ml of erythrocyte-lysis buffer (150 mM NH4Cl, 10 mM KHCO3, 0.1 mM EDTA, H_2_O (pH 7.2-7.4)) were added and incubated at room temperature (RT) for 5 min. Afterward, wash buffer was added to stop the lysis reaction and cells were centrifuged. The cell pellet was washed and meshed again through another 100 µm cell strainer.

### Phenotypic Analysis

Spleen cells were counted and 1.5x10^6^ cells were seeded in a 96-well round-bottom plate. Cells were centrifuged at 400g for 5min at 4°C and the supernatant was discarded. Cells were resuspended in 50 µl extracellular staining mastermix and incubated at RT for 20 min. Cells were fixed and stained intracellularly using the eBioscience FoxP3/transcription factor staining buffer (Life Technologies, Carlsbad, California) according to the manufacturer’s instructions. For sorting, cells were stained extracellularly without any fixation.

For the extracellular staining, we used αLAG-3 (C9B7W, RRID: AB_2871155) BUV737, purchased from BD Biosciences; αLAG-3 (C9B7W, RRID: AB_2572081 & AB_2561517) PE-Dazzle594 & PerCP-Cy5.5, αTIGIT (1G9, RRID: AB_2566573) PE-Dazzle594, αPD-1 (29F.1A12, RRID: AB_2562616) BV605, αTIM-3 (RMT3-23, RRID: AB_2716208) BV711, αCD39 (Duha59, RRID: AB_2563394 & AB_11218603) PE-Cy7 & PE, αCD8α (53-6.7, RRID: AB_2563055 & AB_312745) BV570 & FITC, αCD4 (GK1.5, RRID: AB_2860583) APC/Fire810, αCD44 (IM7, RRID: AB_493713 & AB_830787) Alexa Fluor 700 & PE-Cy7, purchased from BioLegend, San Diego, California. For intracellular staining, αCD3ϵ (145-2C11, RRID: AB_2738278) BUV395 was purchased from BD Biosciences; αGranzyme B (GB11, RRID: AB_2562195) Pacific Blue, αPerforin (S16009A, RRID: AB_2721463) APC, αCTLA-4 (UC10-4B9, RRID: AB_2563063) BV421, αIFN-γ (XMG1.2, RRID: AB_2814432) APC/Fire750 were purchased from BioLegend. Splenocytes of uninfected C57BL/6J mice were used to validate specificity of the stainings (**Figure S1**). Data were acquired on a BD LSR II, BD LSRFortessa, or Cytek Aurora.

### Restimulation Assay

After isolation of spleen cells, 2x10^6^ cells were seeded in a 96-well round-bottom plate and incubated for 5 h with 1 mg/ml Phorbol-myristate-acetate (PMA) and 0.25 mg/ml Ionomycin (Iono) in 200 µl complete RMPI 1640 medium (RPMI 1640 + 5% FCS + 2 mM L-glutamine + 10 mM HEPES + 50 µg/ml Gentamicin) to induce cytokine production. To accumulate cytokines within the cells the medium was also supplemented with Brefeldin A and Monensin (BioLegend) according to the manufacturer’s instructions for the whole incubation period. αCD107a APC antibody (clone 1D4B, RRID: AB_2234505, BioLegend) was added 1:100 to the medium to improve the CD107a staining. BD Cytofix/Cytoperm buffer kit (BD Biosciences) was used for fixation and intracellular staining according to the manufacturers instructions for the restimulation assay.

### Cytotoxicity Assay

OT-I mice (32) were infected with 1x10^5^ iRBC (PbTG (31) 6 days before the day of the assay (see complete workflow of the assay in **Figure S2**). Spleens of two mice were pooled to isolate sufficient numbers of splenocytes. B cells were depleted to reduce sorting time. 20µl PE-labeled αCD19 antibody (clone 6D5, RRID: AB_313643, BioLegend) was added and incubated for 10 min in the dark at RT. The cells were washed, α-PE magnetic beads (Miltenyi, Bergisch Gladbach, Germany) were added and incubated for 10 min. Afterward, the cell suspension was transferred to a magnetic LS-column (Miltenyi). The column was washed twice, the flow-through (containing the CD8^+^ T cells) was washed and stained using αLAG-3 (C9B7W, RRID: AB_2561517) PerCP-Cy5.5, αCD8 (53-6.7, RRID: AB_312745) FITC, and αCD44 (IM7, RRID_ AB_830787) PE-Cy7 (all purchased from BioLegend). The stained cells were sorted into the subsets CD8^+^CD44^+^ LAG-3^neg^, CD8^+^CD44^+^ LAG-3^low^, CD8^+^CD44^+^ LAG-3^high^ and CD8^+^CD44^-^ (whole CD44^-^ cells, without dividing into different levels of LAG-3 expression) on a BD FACS Aria IIIu Cell sorter. After sorting, cells were counted manually, concentrations were adjusted to 4 x 10^6^ cells/ml, and sorted subsets were re-analyzed to ensure high sort purity (**Figure S3**). For the preparation of target cells, whole splenocytes were obtained from sex- and age-matched C57BL/6J mice as described above. The cells were split in half and resuspended in 1 ml complete RPMI. One-half of splenocytes were pulsed with the target peptide for effector cells, using a final concentration of 10 µg/ml SIINFEKL-peptide. Pulsed and unpulsed samples were incubated at 37°C, 5% CO_2_ for 1 h. Subsequently, both samples were washed with PBS, unpulsed cells were stained with eF450, and SIINFEKL-pulsed cells with eF670 tracer dye in PBS (Both Life technologies, Carlsbad, USA). Afterward, cells were washed again, resuspended in complete RPMI, and counted. Cell suspensions were adjusted to 2 x 10^6^ cells/ml and then mixed in a ratio of 1:1, resulting in 1 x 10^6^ pulsed and 1 x 10^6^ unpulsed target cells per ml. Target cells were kept on ice until the effector cells were sorted. Sorted effector cells were washed, counted and adjusted to 4x10^6^ cells/ml. Three different concentrations of effector cells were used in the final experiments: 4 x 10^6^ cells/ml (undiluted), 2 x 10^6^ cells/ml, and 1 x 10^6^ cells/ml. 100 µl (2 x 10^5^ cells) of target cell suspension and 100 µl of pre-diluted effector cells were co-cultured in complete RPMI in a V-bottom plate at 37°C, 5% CO_2_ for 6 h, creating ratios of effector: target cells of 2:1, 1:1 and 0.5:1. One to two technical replicates of every dilution of each sorted subset of effector cells were analyzed. The correct ratio of pulsed to unpulsed target cells was determined by measuring only target cells without effector cells to mitigate any counting errors during the mixing process. After co-culture, the plate was centrifuged at 500g. The supernatant was collected and stored at -20°C until analyzing IFN-γ release. Surviving target cells were identified by staining with ZombieNIR fixable Viability dye (BioLegend) for 15 min at 4°C in the dark. Cells were washed twice with PBS and then fixed with 50 µl Perm/Fix buffer of the FoxP3 transcription buffer staining kit for 20 min. Cells were washed, resuspended in wash buffer, and stored at 4°C until analysis on a BD LSRFortessa. After data acquisition, the proportion of living SIINFEKL-pulsed cells out of all living target cells from the target cell gate was analyzed by following the gating strategy according to **Figure S4**. This ratio is inversely proportional to the percentage of killed pulsed target cells. The proportion is normalized to the mean proportion of SIINFEKL-pulsed cells in the control wells to mitigate any errors occurring by mixing the target cells.

### Cytokine Release

To measure the IFN-γ release, frozen supernatant from co-incubated effector and target cells was analyzed using the DuoSet^®^ ELISA for Mouse IFN-γ (R&D Systems, Minneapolis, Minnesota) according to the manufacturer’s instructions. A linear regression curve was used to calculate the concentration based on the included IFN-γ standard.

### Software and Statistical Analysis

All data were analyzed using GraphPad Prism Version 9. Data were tested for normal distribution using the Anderson-Darling test with a pass for normality of alpha=0.05. A one-way ANOVA with Tukey test or a Friedmann’s test for multiple comparisons was applied, and P-values <0.05 were considered statistically significant. For acquisition of flow cytometry data, we used FACS DIVA (BD LSR II and BD LSRFortessa) and SpectroFlo (Cytek Aurora), respectively. For analysis of flow cytometry data, we used FlowJo Version 10.8. with the UMAP v3.1 (33) for FlowJo Plugin.

## Results

### Expression Levels of LAG-3 on CD8^+^ T Cells Correlate With the Expression of Other Co-Inhibitory Molecules

**Figure 1:**
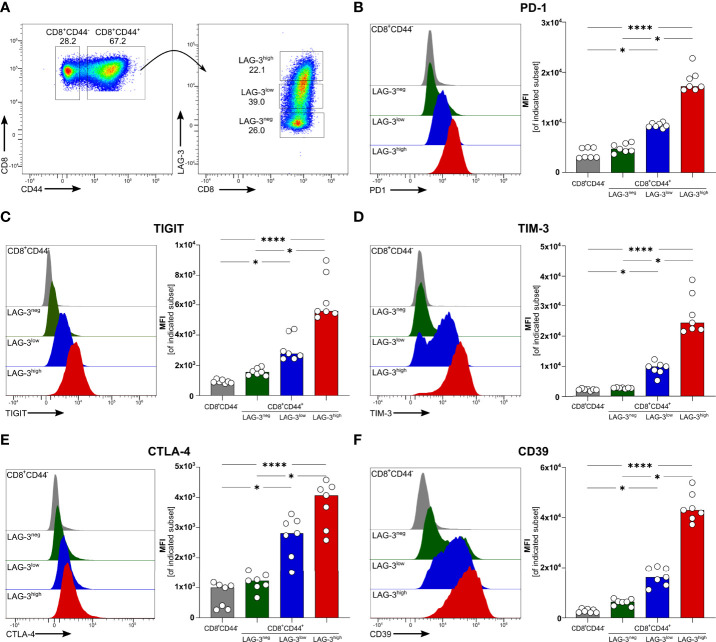
Expression of co-inhibitory molecules and effector molecules correlates with LAG-3 expression. **(A)** Gating strategy and representative examples of dot plots of LAG-3 expression on CD8^+^CD44^+^ T cells. Representative examples of histograms and summarized median fluorescent intensity (MFI) of the co-inhibitory molecules **(B)** PD-1, **(C)** TIGIT, **(D)** TIM-3, **(E)** CTLA-4 and **(F)** CD39 expressed on CD8^+^CD44^+^ T cells expressing different levels of LAG-3 (CD8^+^CD44^+^LAG-3^neg^, CD8^+^CD44^+^LAG-3^low^, CD8^+^CD44^+^LAG-3^high^), compared to expression of those markers on CD8^+^CD44^-^ T cells. Data were analyzed with Friedmann’s test for multiple comparisons. Each dot represents an individual mouse, n = 7 from two independent experiments. P values between ≤ 0.05 (*) and ≤ 0.0001 (****) were considered statistically significant.

We recently described that blood-stage human and experimental malaria is characterized by a strong induction of co-inhibitory molecules on T cells (14). In chronic viral infections the induction of co-inhibitory molecules by T cells dampens their effector function (27). T cells with high expression levels of co-inhibitory molecules are considered to be exhausted (27). Using the PbA model of acute experimental malaria, we compared the functionality of antigen-specific CD8^+^ T cells expressing different levels of co-inhibitory molecules. Whereas CD8^+^CD44^-^ T cells from PbA infected mice lack the expression of any co-inhibitory molecules, PbA-induced CD8^+^CD44^+^ T cells express LAG-3 at variable degree. To further decipher the consequences of variable expression levels of co-inhibitory molecules on T cell function, we distinguish between CD8^+^CD44^+^ T cells expressing no (LAG-3^neg^), low (LAG-3^low^) and high levels of LAG-3 (LAG-3^high^) (**Figure 1A**; **Figure S5**). Interestingly, increasing expression levels of LAG-3 correlates with increasing co-expression of other co-inhibitory molecules. CD8^+^CD44^+^LAG-3^low^ T cells express intermediated levels of PD-1, TIGIT, TIM-3 and Cytotoxic T-Lymphocyte-Associated Protein 4 (CTLA-4), whereas CD8^+^CD44^+^LAG-3^high^ T cells exhibit statistically significant higher median fluorescence intensities of these co-inhibitory molecules compared to CD8^+^CD44^+^LAG-3^low^ and CD8^+^CD44^+^LAG-3^neg^ T cells (**Figures 1B–E**). Also, CD39, an ecto-nucleosidase implicated in T cell exhaustion (21, 22), correlates with the expression of LAG-3 (**Figure 1F**).

### High Expression Levels of Co-Inhibitory Molecules Are not Associated With Exhaustion but Rather Increased Function

**Figure 2:**
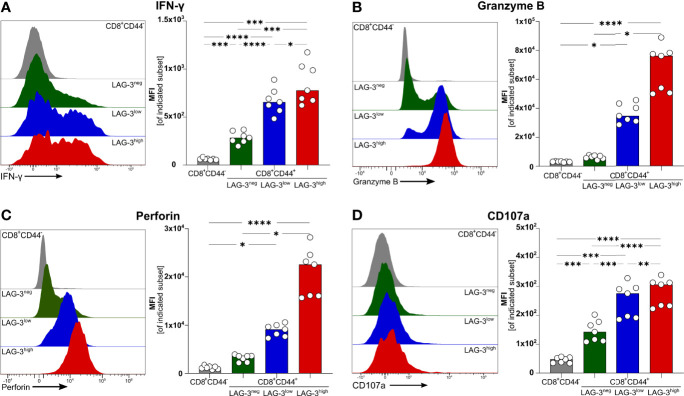
Expression of effector molecules correlates with LAG-3 expression. Exemplary histograms and MFI of **(A)** IFN-γ, **(B)** Granzyme B, **(C)** Perforin and **(D)** CD107a expressed by CD8^+^CD44^+^ cells expressing different levels of LAG-3 (CD8^+^CD44^+^LAG-3^neg^, CD8^+^CD44^+^LAG-3^low^, CD8^+^CD44^+^LAG-3^high^) and by CD8^+^CD44^-^ T cells. Cells were isolated from the spleens of C57BL/6J infected with PbA 6 days post-infection. For B&C, cells were stained directly *ex vivo.* For A&D, cells were stained after 5 h restimulation with PMA/Iono in the presence of Brefeldin A and Monensin. Data were analyzed with Tukey test for multiple comparisons (A&D) or Friedmann’s test (B&C). Each dot represents an individual mouse, n=7. P values ≤ 0.05 (*), ≤ 0.01 (**), ≤ 0.001 (***) or ≤ 0.0001 (****) were considered statistically significant.

To verify if effector functions of malaria-induced CD8^+^ T cells are influenced by the expression of co-inhibitory molecules, we analyzed the intracellular expression of the pro-inflammatory cytokine Interferon γ (IFN-γ) as well as Granzyme B (GrzB) and Perforin, both of which are key effector molecules for the cytotoxic function of CD8^+^ T cells. Interestingly, IFN-γ, GrzB, and Perforin were found to be higher expressed with increasing expression of co-inhibitory molecules (**Figures 2A–C**). Similarly, the surface expression of the degranulation marker CD107a increases with the expression of co-inhibitory molecules (**Figure 2D**). Thus, malaria-induced CD8^+^ T cells co-express LAG-3, PD-1, TIGIT, TIM-3, CTLA-4, and CD39 but similarly express more effector molecules compared to those expressing lower levels of co-inhibitory molecules.

### Malaria-Induced CD8^+^ T Cells Expressing Higher Levels of Co-Inhibitory Molecules Are More Cytotoxic

**Figure 3:**
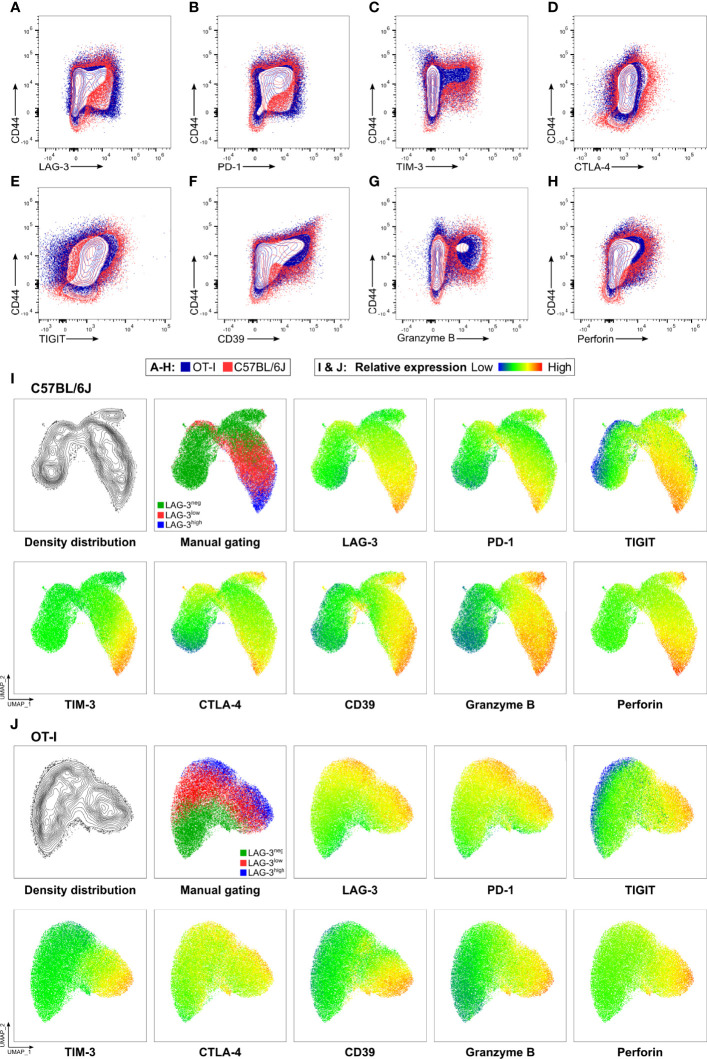
OT-I derived CD8^+^CD44^+^ T cells express co-inhibitory and effector molecules in a similar fashion to C57BL/6J derived CD8^+^CD44^+^ T cells. Exemplary contour showing expression of **(A)** LAG-3, **(B)** PD-1, **(C)** TIM-3, **(D)** CTLA-4, **(E)** TIGIT, **(F)** CD39, **(G)** GrzB and **(H)** Perforin on CD8^+^ T cells relative to CD44 expression. Cells were obtained from the spleens of C57BL/6J (red), and OT-I (blue) mice infected with PbTG 6 days past infection. **(I, J)** Representative UMAP clustering of CD44^+^CD8^+^ T cells isolated from C57BL/6J and OT-I mice infected with PbTG 6 days post infection showing the density distribution within the plot; classification into CD44^+^LAG-3^neg^, CD44^+^LAG-3^low^ and CD44^+^LAG-3^high^ as used throughout the manuscript and relative expression of indicated molecules. Sampled were downsampled to 40.000 cells. UMAP parameters: Distance: Euclidean, Nearest Neighbours: 15, Minimum Distance: 0.5, Number of Components: 2, UMAP v3.1 for FlowJo.

Next, we determined the cytotoxic capacity of CD8^+^ T cells rich in co-inhibitory molecules in experimental malaria. Since the function of T cells is not only regulated by co-stimulation and co-inhibition but also by the broad spectrum of affinities of their individual T cell receptor (TCR) to their cognate MHC-class I antigen complex, we used OT-I TCR-TG mice with a transgenic SIINFEKL-specific MHC I on CD8^+^ T cells (OT-I mice) (32). Infecting these mice with PbA parasites expressing OVA (PbTG) (31) allowed us to induce a strong activation of transgenic SIINFEKL-specific CD8^+^ T cells, thereby eliminating the influence of the variety of different TCR signaling strength on T cell function. Similar to wild type C57BL/6J mice (**Figure 2**), activated CD8^+^CD44^+^ T cells from OT-I mice express high levels of the co-inhibitory molecules LAG-3, PD-1, TIM-3, CTLA-4 and TIGIT, the ecto-nucleosidase CD39 and the effector molecules Perforin and GrzB (**Figures 3A–H**, **Figure S5**). However, UMAP analysis of CD8^+^CD44^+^ T cells from C57BL/6J mice and OT-I mice revealed that co-expression of co-inhibitory molecules is less pronounced in the later (**Figures 3I, J**). Nevertheless, this analysis confirmed that T cells expressing GrzB and Perforin in the CD8^+^CD44^+^LAG-3^high^ compartment do co-express the aforementioned molecules. This finding was corroborated by a correlation analysis showing statistically significant co-expression in CD8^+^ T cells from C57BL/6J and OT-I mice (**Figure S6**). To investigate the influence of the expression of co-inhibitory molecules on the cytotoxic function of malaria-induced CD8^+^ T cells, these were sorted according to their LAG-3 expression (**Figure S3A**). Re-analysis of sorted cells revealed distinct populations of CD8^+^CD44^-^, CD8^+^CD44^+^LAG-3^neg^, CD8^+^CD44^+^LAG-3^low^ and CD8^+^CD44^+^LAG-3^high^ (**Figure S3B**), which we then analyzed in a newly developed cytotoxicity assay. To this end, *in vivo* activated, sorted OT-I cells were co-cultured with SIINFEKL-pulsed target cells and specific killing of those target cells was determined by flow cytometry (**Figure S4**; a schematic overview of the workflow is depicted in **Figure S2**).

**Figure 4:**
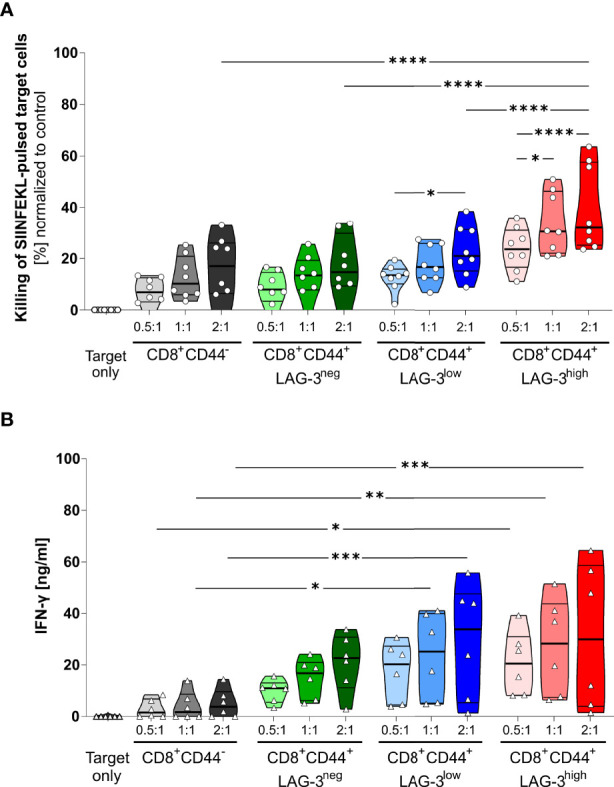
LAG-3 expression correlates with cytotoxic capabilities of CD8^+^ T cells. **(A)** Killing of SIINFEKL (SII)-pulsed target cells by CD8^+^CD44^+^ T cells expressing different levels of LAG-3 compared to CD8^+^CD44^-^ T cells in a dose dependent manner. SIINFEKL-pulsed target cells obtained from C57BL/6J mice were co-incubated with indicated sorted CD8^+^ T cells in indicated ratios for 6 hours. CD8^+^ T cells were obtained from OT-I mice infected with PbTG 6 days prior to T cell isolation. **(B)** Secretion of IFN-γ of the above-mentioned CD8^+^ T cells subsets from the supernatant of the cytotoxicity assay. Each dot represents the mean of up to two technical replicates. Data were tested for normal distribution with the Anderson-Darling test with a pass for normality of alpha=0.05. ANOVA with Tukey test for multiple comparisons. P= * < 0.05, ** < 0.01, *** < 0.001, **** < 0.0001, **(A)** n = 8, **(B)** n = 6. All groups showed significant (P < 0.05) killing compared to the target only group; these significances are not shown to avoid overloading the figure.

The proportion of specifically killed target cells increases in CD8^+^CD44^+^LAG-3^low^ and CD8^+^CD44^+^LAG-3^high^ populations compared to their CD8^+^CD44^-^ and CD8^+^CD44^+^LAG-3^neg^ counterparts (**Figure 4A**). Furthermore, the target cell specific killing depends on the dose of effector T cells. Also, CD8^+^CD44^+^LAG-3^high^ effector cells diminish significantly more pulsed target cells than the CD8^+^CD44^+^LAG-3^low^ effector cell subset, indicating increased cytotoxic capacity with increasing levels of expression of co-inhibitory molecules. This effect is also visible comparing lower effector to target cell ratios.

These data were further confirmed by analyzing the IFN-γ production of the CD8^+^CD44^+^ T cells distinguished on their LAG-3 expression (**Figure 4B**). While CD8^+^CD44^-^ T cells show low amounts of IFN-γ production, increasing levels of LAG-3 expression correlate with increasing levels of IFN-γ production, implying that cells expressing more co-inhibitory molecules show higher cytotoxic capacity.

## Discussion

Blood-stage malaria is characterized by overwhelming activation of CD4^+^ as well as CD8^+^ T cells, both of which secrete pro-inflammatory cytokines. These T cells confer protection by activating macrophages capable of clearing infected erythrocytes, especially in the spleen and the liver (6–8). In addition, CD8^+^ T cells were shown to directly lyse *P. vivax* infected reticulocytes (5). However, they also contribute to activation of endothelial cells which enhances sequestration of infected erythrocytes and cross-presentation of parasitic antigen (34), a hallmark of severe malaria (35–37). Recent data also provide evidence that in experimental and human malaria, CD8^+^ T cells contribute to the dissolution of the endothelial cell layer and thereby loss of vascular integrity through their cytotoxic capacity (38). Along this line, we have recently shown that GrzB secretion by CD8^+^ T cells correlates with severity in acute human malaria (11) and CD8^+^Grzb^+^ T cells adhere/accumulate on the brain’s vasculature of fatal cerebral malaria (39). These data support the use of the experimental *P. berghei* model for deducing the immunopathology in human malaria. Therefore, tight regulation of T cell function is crucial to ensure efficient parasite clearance and/or control without overwhelming inflammation being a prerequisite for severe malaria. We have recently shown that blood-stage malaria in experimental models but also in human malaria is accompanied by an induction of co-inhibitory molecules like PD-1, LAG-3, and TIM-3 on CD4^+^ as well as CD8^+^ T cells.

We showed malaria-specific co-inhibitory rich CD4^+^ T cells to be capable of suppressing the activation of naïve T cells and might regulate the amount of T cell activation in acute malaria (14, 16, 40). Interestingly, we also found CD8^+^ T cells with a similar co-inhibitory molecule expression with the same suppressive capacity (14). Therefore, induction of co-inhibitory molecules in acute malaria delineate T cells with functional properties of type 1 regulatory T cells (Tr1 cells) (41). However, expression of co-inhibitory molecules on CD8^+^ T cells in settings of prolonged antigen exposure like chronic viral infections or cancer was shown to limit T cell function leading to a state often referred to as T cell exhaustion (27). Blocking co-inhibitory molecules by antibodies was shown to, at least partially, revert T cell function, often having a great beneficial effect in cancer therapy (26, 30). The therapeutic effect of blocking co-inhibitory molecules in chronic viral diseases is less well studied. Increasing the function of antigen-specific CD8^+^ T cells might help to clear persisting virus (42). However, since the viral load in specific organs can be very high and many cells in the respective tissue might be affected, unleashing the function of exhausted CD8^+^ T cells might have detrimental effects on organ function. Interestingly, it was shown that a blockade of PD-1 in experimental malaria increases T cell memory (43). It was also shown in an LCMV model that LAG-3^+^CD8^+^ T cells have a disadvantage in memory formation compared to CD8^+^ T cells from LAG-3-deficient mice (44). Thus, an expression of co-inhibitory molecules on CD8^+^ T cells as found in acute malaria might restrict memory formation.

In hepatocellular carcinoma, T cell exhaustion is associated with high expression of PD-1 on CD8^+^ T cells. The extent of exhaustion correlates with increased expression levels of PD-1 and co-expression of other co-inhibitory molecules (45). The reasons mentioned above prompted us to study the regulation of CD8^+^ T cells in acute malaria in more detail. We hypothesized that CD8^+^ T cells expressing high levels of multiple co-inhibitory molecules are restricted in their functionality, whereas CD8^+^ T cells express less co-inhibitory molecules comprising strongly activated T effector cells. However, studying the function of bulk malaria-specific CD8^+^ T cells in relation to expression with co-inhibitory molecules has several flaws.

Activation of CD8^+^ T cells is dependent on multiple factors like co-stimulation and TCR affinity to antigen MHC class I complexes. Due to the vast amount of different plasmodial antigens, the resulting CD8^+^ T cell response is inherently heterogenic. Therefore, a correlation of co-inhibitory molecules and T effector function remains elusive. To circumvent this, we employed a transgenic system using OVA-expressing PbTG to activate SIINFEKL-specific CD8^+^ T cells from OT-I mice. This ensures that all activated T cells express an identical T cell receptor. Using this system, we found that also activated transgenic CD8^+^CD44^+^ T cells expressing high levels of LAG-3, also express high levels of other co-inhibitory molecules like PD-1, TIGIT, TIM-3 and CTLA-4. In addition, the strongest expression of CD39, a molecule often associated with exhaustion, was found on CD8^+^CD44^+^LAG-3^high^ T cells. CD8^+^CD44^+^LAG-3^low^ T cells, in contrast, exhibit a weaker expression of all aforementioned co-inhibitory molecules, whereas activated CD8^+^CD44^+^LAG-3^neg^ T cells do express only low levels of indicated co-inhibitory molecules. Using UMAP-clustering and correlation analysis, we showed that the expression-pattern of co-inhibitory molecules is similar between malaria-induced CD8^+^ T cells from OT-I and C57BL/6J mice. Interestingly, less LAG-3^high^ OT-I T cells express GrzB and Perforin compared to those T cells from C57BL/6J mice. However, the majority of GrzB and Perforin producing T cells are still within the LAG-3^high^ compartment and do co-express the other co-inhibitory molecules thereby demonstrating a similar expression pattern to CD8^+^CD44^+^ T cells found in PbA-infected C57BL/6J mice. Those differences might be explained by different TCR-affinities and/or kinetics of antigen-expression of plasmodial antigens and the recombinant expressed model antigen. This allows us to compare the function of activated CD8^+^ T cells with a uniform expression of low, medium and high expression of multiple co-inhibitory molecules.

Interestingly, after restimulation, the effector function of CD8^+^ T cells positively correlates with the expression of co-inhibitory molecules. CD8^+^CD44^+^LAG-3^high^ cells not only produced the highest amount of IFN-γ but produced also the highest amount of the cytotoxic molecules GrzB and Perforin. Furthermore, CD107a, a marker for degranulation, showed the highest expression on CD8^+^CD44^+^LAG-3^high^ cells, indicating their increased cytotoxic potential. To further evaluate their cytotoxic capacity, we purified activated CD8^+^CD44^+^ T cells according to their expression level of co-inhibitory molecules. Using a flow cytometry-based cytotoxic assay with differentially labeled SIINFEKL-pulsed and unpulsed target cells, we can corroborate the finding of an increase of cytotoxic function with increased expression of co-inhibitory molecules. This finding clearly demonstrates that even the high expression of multiple co-inhibitory molecules in acute malaria is not associated with decreased effector function or exhaustion. In contrast, it appears that in this acute setting of inflammation, the expression of co-inhibitory molecules delineates CD8^+^ T cells with the highest effector function. Consistent with this is the recent finding that activation of the PD1-pathway by treatment with programmed death-ligand 1 (PD-L1) fusion proteins may be protective in a model of lethal experimental cerebral malaria (CM) due to inhibition of CD8^+^ T cells derived cytotoxicity (46). It was recently shown that dampening the function of CD8^+^ T cells by IL-10-producing NK cells can prevent experimental cerebral malaria (47). However, it remains unclear if the high expression of multiple co-inhibitory molecules on malaria-specific CD8^+^ T cells might have the potential to dampen their function. We could think of several reasons that might contribute to the absence of exhaustion. The strong inflammatory microenvironment with a vast amount of plasmodial antigens, toll-like receptor (TLR)-agonistic molecules, pro-inflammatory cytokines, high expression of co-stimulatory molecules, and the lack of co-inhibitory ligand interaction might shift the balance to co-stimulation rather than co-inhibition. In addition, several studies have shown that the duration of antigen persistence is critical for establishing exhaustion. Therefore, regarding the highly dynamic kinetics of T cell activation in acute malaria, expression of co-inhibitory molecules might not exert their potential suppressive effects. In conclusion, the high expression of multiple co-inhibitory molecules during acute antigen exposure in blood stage malaria on CD8^+^ T cells is associated with enhanced cytokine production and cytotoxic activity instead of decreased function. These results are relevant as they provide novel insights into T cell regulation and the function of co-inhibitory molecules in malaria.

## Data Availability Statement

The original contributions presented in the study are included in the article/**Supplementary Material**. Further inquiries can be directed to the corresponding author.

## Ethics Statement

The animal study was reviewed and approved by Behörde für Justiz und Verbraucherschutz, Hansestadt Hamburg, Germany.

## Author Contributions

JB, MR and TJ designed the experiments. JB and AH conducted the experiments. JB, MR and TJ analyzed and interpreted the data. JB, MR, AH and TJ wrote the manuscript and prepared the figures. All authors reviewed the manuscript.

## Funding

This project has been funded by the Deutsche Forschungsgemeinschaft SFB841 (TJ) and Deutsches Zentrum für Infektionsforschung DZIF (TJ and AH).

## Conflict of Interest

The authors declare that the research was conducted in the absence of any commercial or financial relationships that could be construed as a potential conflict of interest.

## Publisher’s Note

All claims expressed in this article are solely those of the authors and do not necessarily represent those of their affiliated organizations, or those of the publisher, the editors and the reviewers. Any product that may be evaluated in this article, or claim that may be made by its manufacturer, is not guaranteed or endorsed by the publisher.

## Supplementary Material

Supplementary Figure 1 Representative stainings demonstrating upregulation of co-inhibitory and effector molecules upon infection. To validate the specificity of the stainings, splenocytes isolated from an uninfected (termed naïve, grey) and an infected C57BL/6J were stained and compared in an overlay. **(A)** *Ex vivo* stainings of LAG-3, PD-1, TIGIT, TIM-3, CTLA-4, CD39, Granzyme B and Perforin. **(B)** IFN-γ and CD107a stainings after restimulation with PMA/Ionomycin.

Supplementary Figure 2 Workflow of the cytotoxicity assay. OT-I derived splenocytes cells are depleted of B cells and sorted to obtain the subsets of interest. In the meantime, splenocytes from a naïve C57BL/6J are isolated and a portion is pulsed with SIINFEKL. The C57BL/6J derived splenocytes are labelled with their respective tracer dye, mixed and co-incubated with the sorted CD8^+^ T cells. Killing of SIINFEKL-pulsed target cells is measured *via* flow cytometry and used as a readout to determine cytotoxic capacity of the different CD8^+^ T cell subsets.

Supplementary Figure 3 Sorting for CD8^+^ T cells. **(A)** Gating strategy to sort CD8^+^CD44^+^LAG-3^neg,low,high^ and CD8^+^CD44^-^ cells isolated from OT-I mice infected with PbTG 6 days after infection. PE-CD19 labelled cells were removed using α-PE magnetic beads prior to sorting to reduce sorting time. **(B)** Re-analysis of sorted cells, gated on single CD8^+^ T cells to control the purity of sorted populations.

Supplementary Figure 4 Analysis of cytotoxicity assay. **(A–D)** Gating strategy to analyse the proportion of living SIINFEKL(SII)-loaded target cells compared to unloaded target cells after incubation with different CD8^+^ T cell subsets obtained from an OT-I mice infected with PbTG. Effector cells are excluded from the target cells through the lack of either eF450 or eF670 tracer dye. **(E–I)** Representative target cell gates of **(E)**Target cells only and target cells incubated with **(F)** CD8^+^CD44^-^ **(G)** CD8^+^CD44^+^LAG-3^neg^, **(H)** CD8^+^CD44^+^LAG-3^low^ and **(I)** CD8^+^CD44^+^LAG-3^high^ in the highest ratio of effector: target cells of 2:1. A lower percentage of SII-loaded cells relative to the control indicates the higher cytotoxic capacity of the respective subset of CD8^+^ T cells.

Supplementary Figure 5 Expression of co-inhibitory molecules and effector molecules correlate with LAG-3 expression in OT-I mice. MFI of the co-inhibitory molecules PD-1, TIGIT, TIM-3, CTLA-4, CD39 and effector molecules CD107a, Granzyme B, Perforin and IFN-γ expressed on CD8^+^CD44^+^ T cells expressing different levels of LAG-3 (CD8^+^CD44^+^LAG-3^neg^, CD8^+^CD44^+^LAG-3^low^, CD8^+^CD44^+^LAG-3^high^) compared to expression of those markers on CD8^+^CD44^-^ T cells isolated from OT-I mice infected with PbTG. Data were analyzed with Friedmann’s test for multiple comparisons. Each dot represents an individual mouse, n=8 from two independent experiments. P values between ≤ 0.05 (*) and ≤ 0.0001 (****) were considered statistically significant.

Supplementary Figure 6 Correlation of analyzed markers on C57BL/6J ad OT-I mice. Correlation of markers analyzed from the *ex vivo* staining on CD8^+^ T cells isolated from infected C57BL/6J and OT-I mice. **(A, B)** Representative stainings in which all markers are plotted against each other. The accompanying correlation matrix showing **(C, D)** Pearson´s correlation coefficient between the indicated markers was calculated in GraphPad Prism based on 25.000 CD8^+^ T cells. P values: <0.0001 (****).
