# Supplementary figures and images for: Increased Expression of Multiple Co-Inhibitory Molecules on Malaria-Induced CD8+ T Cells Are Associated With Increased Function Instead of Exhaustion
Source: Front Immunol. 2022 Jul 7;13:878320. doi: 10.3389/fimmu.2022.878320 (PMC9301332; doi:10.3389/fimmu.2022.878320)

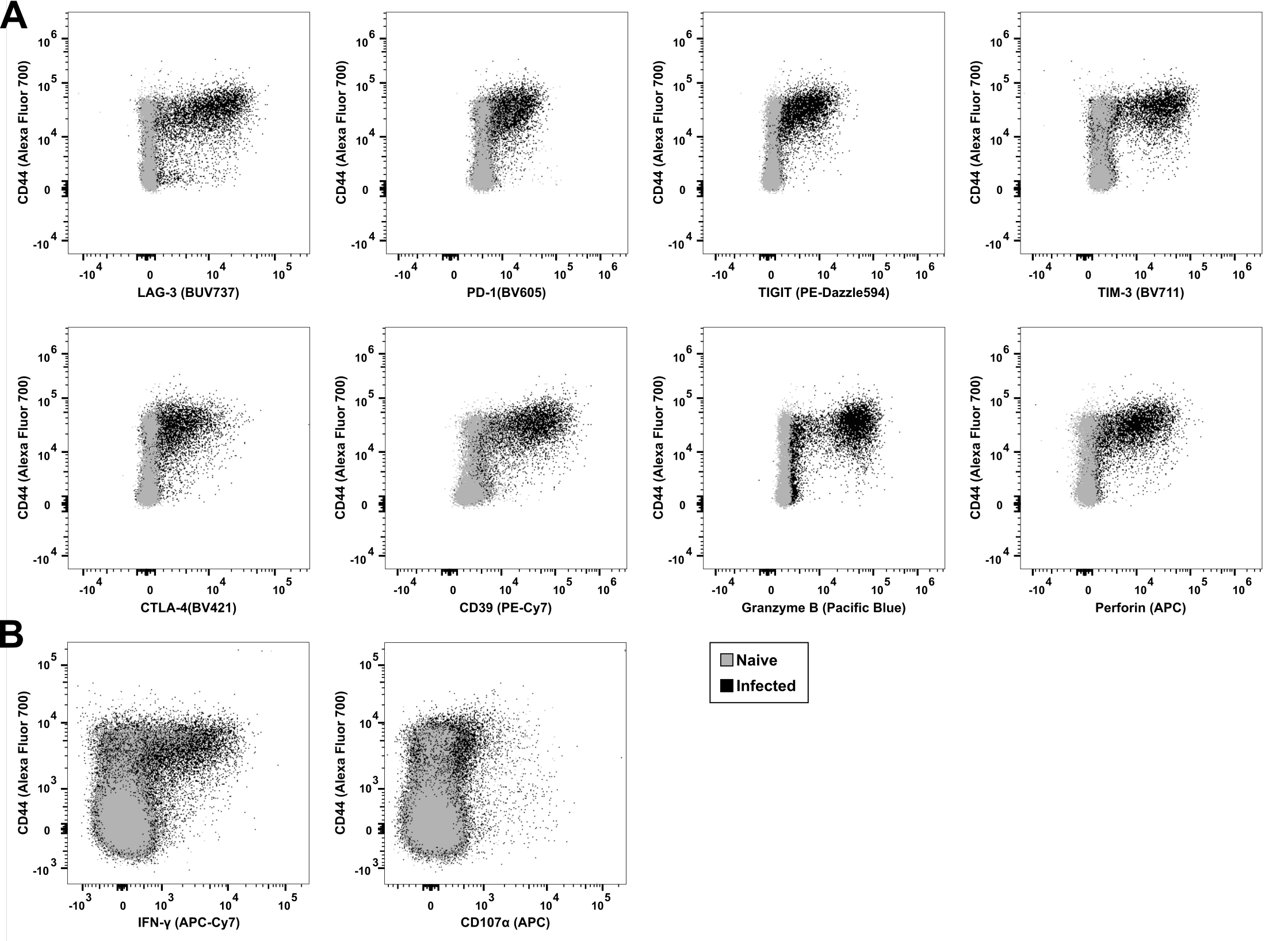

Supplement: Supplementary Figure 1 — Representative stainings demonstrating upregulation of co-inhibitory and effector molecules upon infection. To validate the specificity of the stainings, splenocytes isolated from an uninfected (termed naïve, grey) and an infected C57BL/6J were stained and compared in an overlay. (A) Ex vivo stainings of LAG-3, PD-1, TIGIT, TIM-3, CTLA-4, CD39, Granzyme B and Perforin. (B) IFN-γ and CD107a stainings after restimulation with PMA/Ionomycin. [file Image_1.tif]

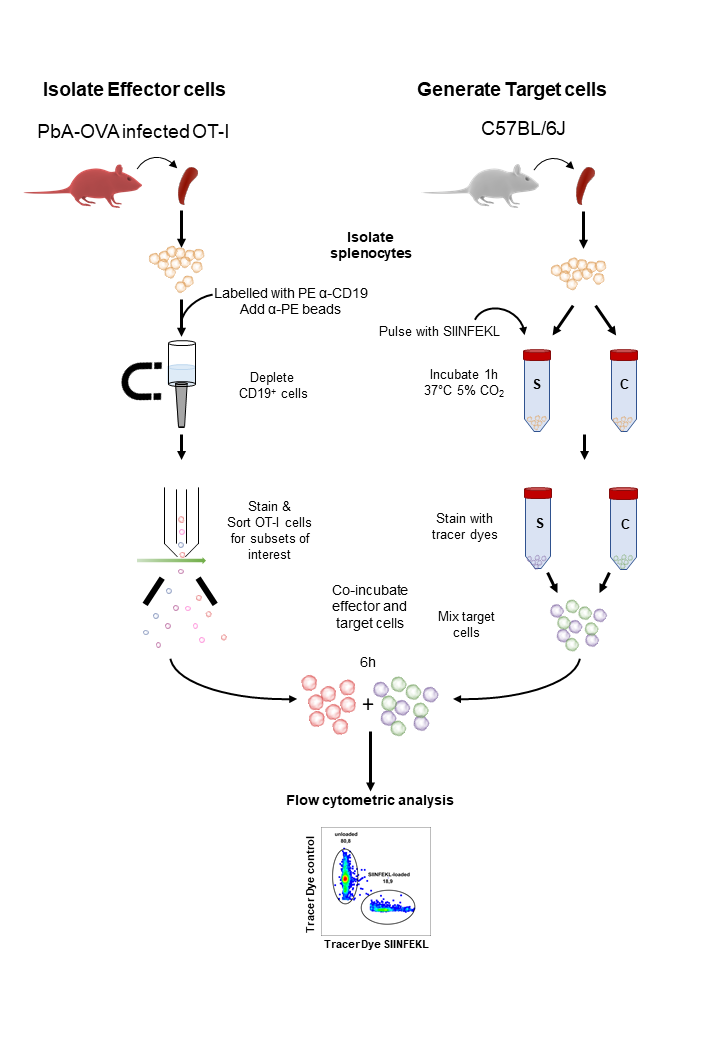

Supplement: Supplementary Figure 2 — Workflow of the cytotoxicity assay. OT-I derived splenocytes cells are depleted of B cells and sorted to obtain the subsets of interest. In the meantime, splenocytes from a naïve C57BL/6J are isolated and a portion is pulsed with SIINFEKL. The C57BL/6J derived splenocytes are labelled with their respective tracer dye, mixed and co-incubated with the sorted CD8+ T cells. Killing of SIINFEKL-pulsed target cells is measured via flow cytometry and used as a readout to determine cytotoxic capacity of the different CD8+ T cell subsets. [file Image_2.tif]

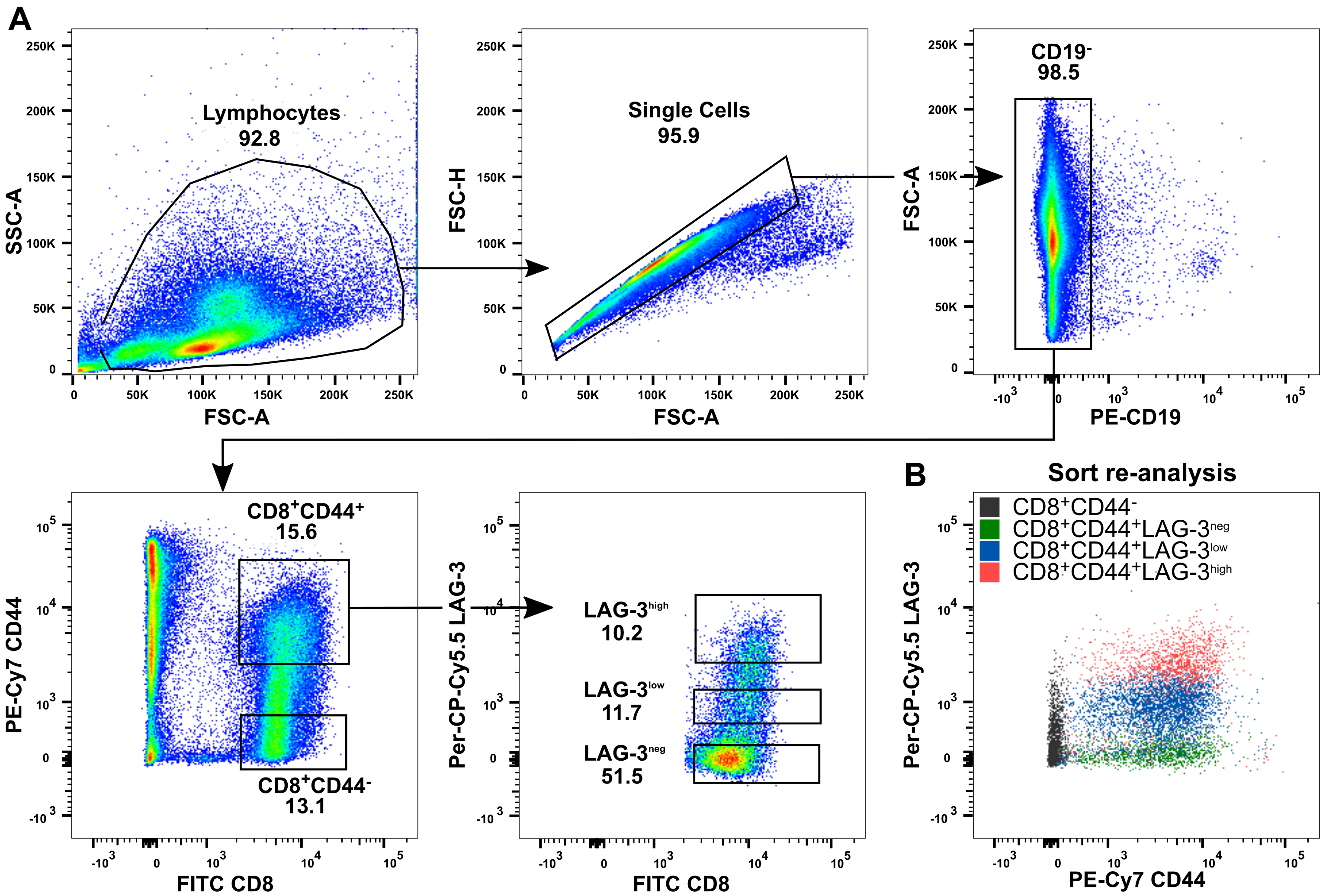

Supplement: Supplementary Figure 3 — Sorting for CD8+ T cells. (A) Gating strategy to sort CD8+CD44+LAG-3neg,low,high and CD8+CD44- cells isolated from OT-I mice infected with PbTG 6 days after infection. PE-CD19 labelled cells were removed using α-PE magnetic beads prior to sorting to reduce sorting time. (B) Re-analysis of sorted cells, gated on single CD8+ T cells to control the purity of sorted populations. [file Image_3.tiff]

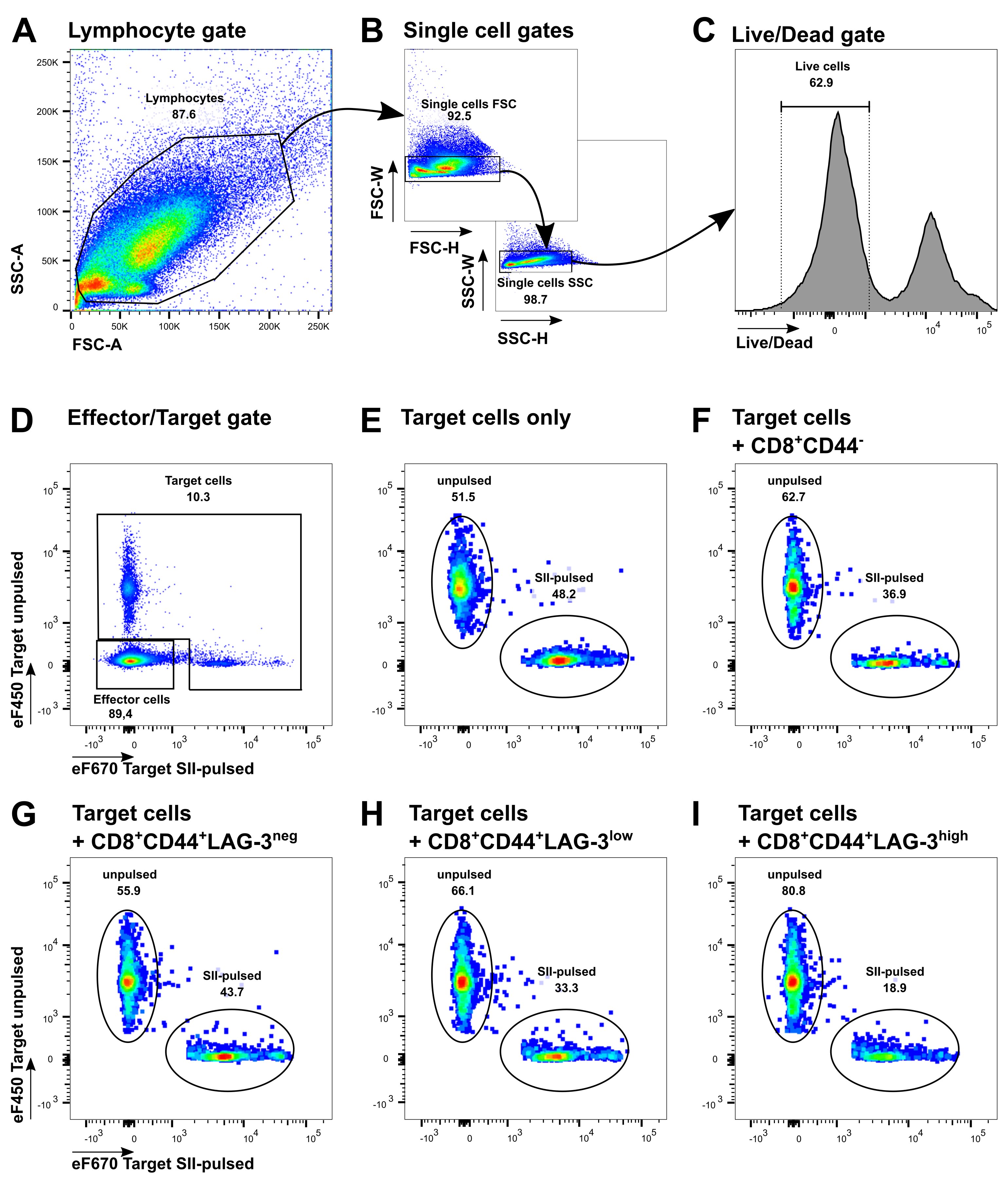

Supplement: Supplementary Figure 4 — Analysis of cytotoxicity assay. (A–D) Gating strategy to analyse the proportion of living SIINFEKL(SII)-loaded target cells compared to unloaded target cells after incubation with different CD8+ T cell subsets obtained from an OT-I mice infected with PbTG. Effector cells are excluded from the target cells through the lack of either eF450 or eF670 tracer dye. (E–I) Representative target cell gates of (E)Target cells only and target cells incubated with (F) CD8+CD44- (G) CD8+CD44+LAG-3neg, (H) CD8+CD44+LAG-3low and (I) CD8+CD44+LAG-3high in the highest ratio of effector: target cells of 2:1. A lower percentage of SII-loaded cells relative to the control indicates the higher cytotoxic capacity of the respective subset of CD8+ T cells. [file Image_4.tiff]

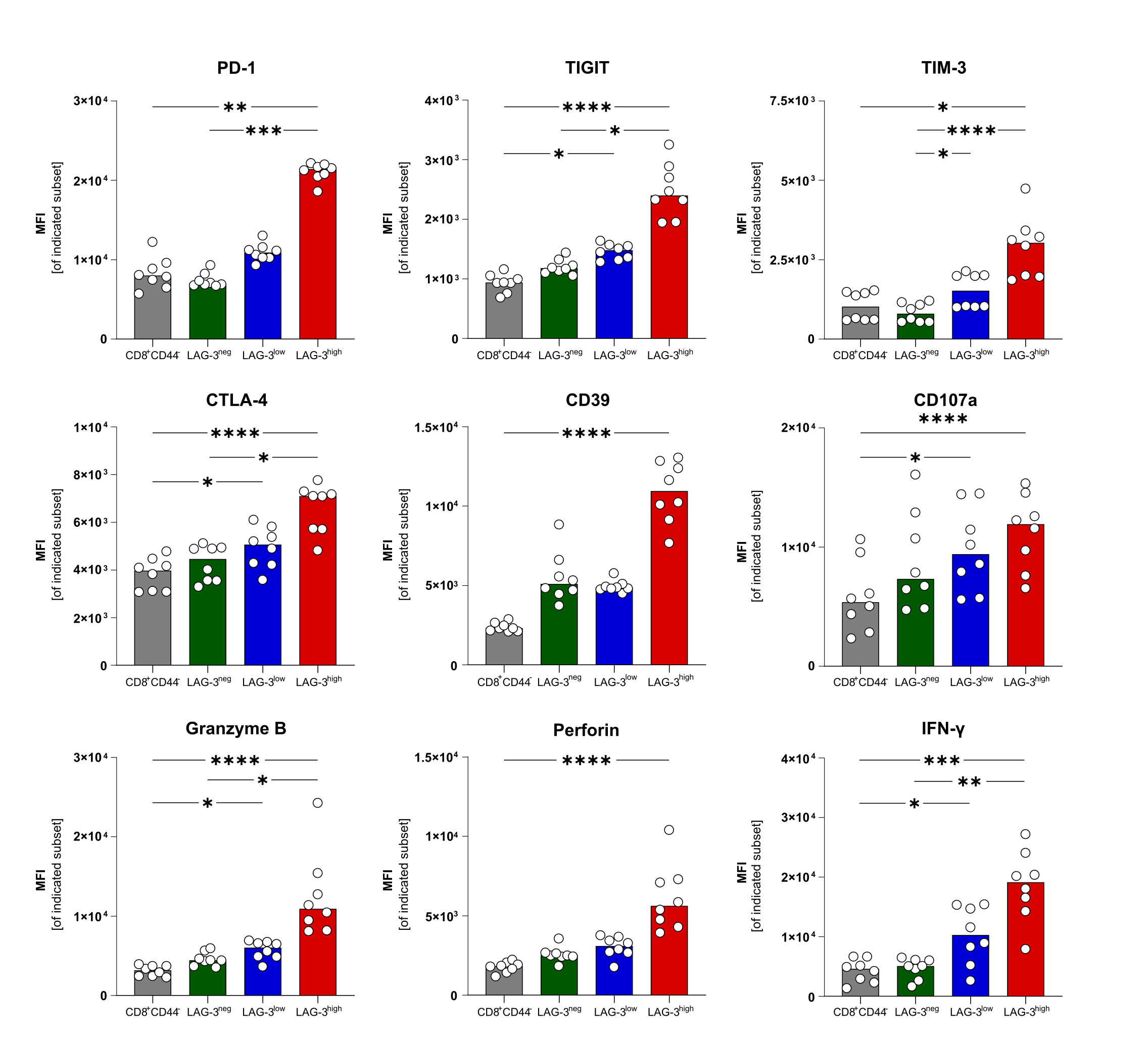

Supplement: Supplementary Figure 5 — Expression of co-inhibitory molecules and effector molecules correlate with LAG-3 expression in OT-I mice. MFI of the co-inhibitory molecules PD-1, TIGIT, TIM-3, CTLA-4, CD39 and effector molecules CD107a, Granzyme B, Perforin and IFN-γ expressed on CD8+CD44+ T cells expressing different levels of LAG-3 (CD8+CD44+LAG-3neg, CD8+CD44+LAG-3low, CD8+CD44+LAG-3high) compared to expression of those markers on CD8+CD44- T cells isolated from OT-I mice infected with PbTG. Data were analyzed with Friedmann’s test for multiple comparisons. Each dot represents an individual mouse, n=8 from two independent experiments. P values between ≤ 0.05 (*) and ≤ 0.0001 (****) were considered statistically significant. [file Image_5.tif]

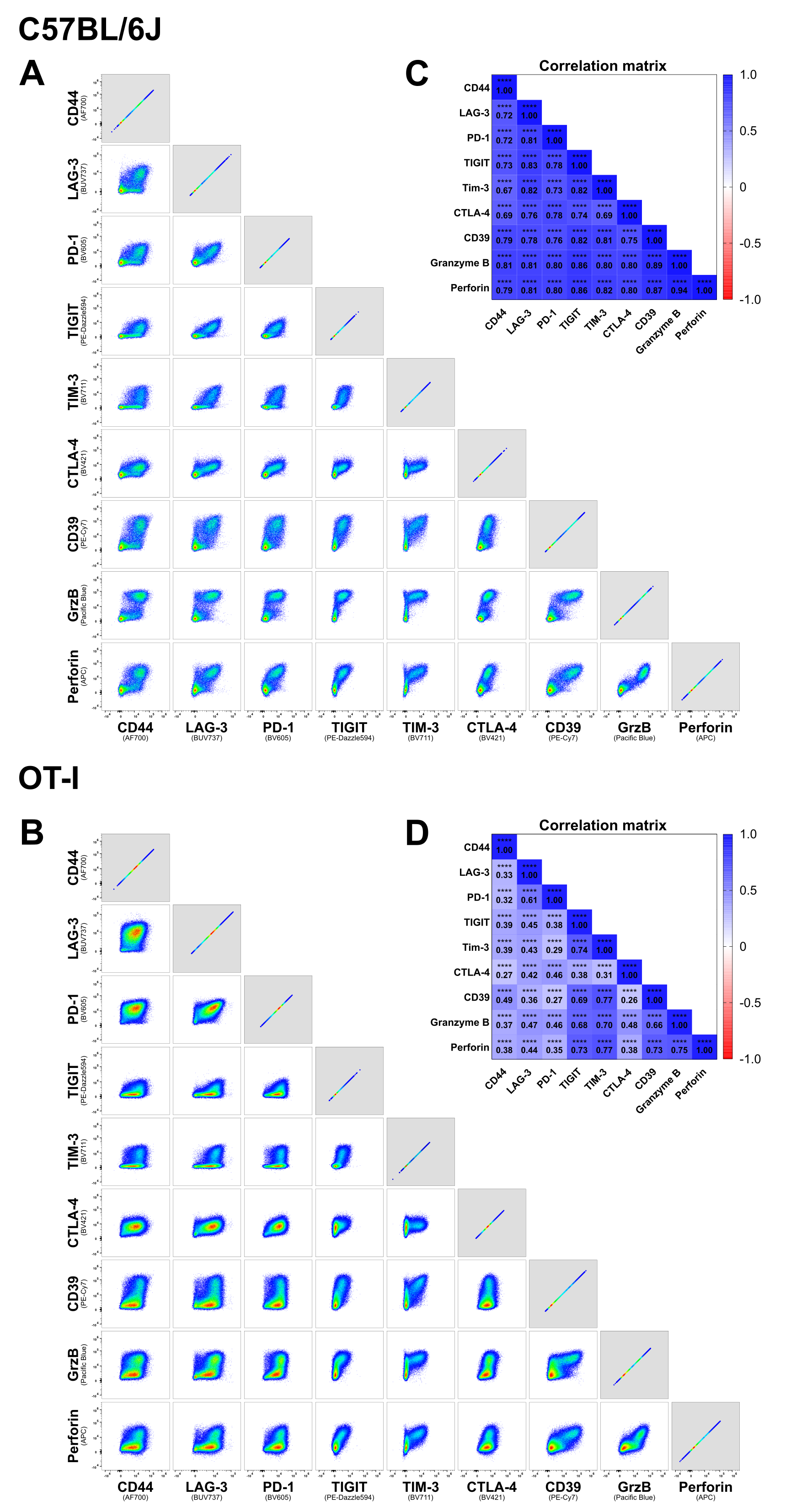

Supplement: Supplementary Figure 6 — Correlation of analyzed markers on C57BL/6J ad OT-I mice. Correlation of markers analyzed from the ex vivo staining on CD8+ T cells isolated from infected C57BL/6J and OT-I mice. (A, B) Representative stainings in which all markers are plotted against each other. The accompanying correlation matrix showing (C, D) Pearson´s correlation coefficient between the indicated markers was calculated in GraphPad Prism based on 25.000 CD8+ T cells. P values: <0.0001 (****). [file Image_6.tiff]
